# Statistical modeling of mutagenic azo dye adsorption on bagasse activated carbon

**DOI:** 10.1038/s41598-025-04240-9

**Published:** 2025-06-20

**Authors:** Basma M. Ismail, Ahmed M. Zayed, Mahmoud A. Roshdy, Mohamed Abdel Rafea, Fathy M. Mohamed

**Affiliations:** 1 Water and Environment Department, Faculty of Earth Sciences, Beni-Suef University, Beni-Suef City, Egypt; 2 Applied Mineralogy and Water Research Lab, Department of Geology, Faculty of Science, Beni-Suef University, Beni-Suef City, Egypt; 3 Department of Physics, College of Science, Imam Mohammad Ibn Saud Islamic University (IMSIU), 11623 Riyadh, Kingdom of Saudi Arabia

**Keywords:** Agro-residue, Activated charcoal, Metal-complex dyes, Removal, Statistical tools, Ecology, Environmental sciences, Health care, Chemistry

## Abstract

The current study investigates the development and characterization of sustainable activated carbons (ACs) via chemo-thermal activation from the hull and core of sugarcane bagasse as a viable and renewable substitute for commercial ACs. Characterize ACs using XRD, FTIR, SEM, etc. The sorption kinetics of methylene blue (MB) onto AC(H) were well described by a pseudo-second-order model. Also, the controlling step in the MB sorption process was related to several intervening diffusion sorts, including intra-particle ones. The MB equilibrium data were also analyzed using linear and non-linear forms of Langmuir, Freundlich, and Temkin isotherms, revealing a better fit of Langmuir, with R^2^ values > 0.97 in both modes. With adsorption capacities (q_max_ = 357.14 and 389.4 mg/g) in linear and non-linear modes, orderly. The activation energy (EDR) of 550.8 and 2500 J/mol in non-linear and linear further supports the dominance of chemisorption, implying the formation of chemical bonds between the MB and the functional groups present in the sorbent material. The spontaneous and exothermic nature of the MB sorption process at 291–323 K was confirmed by the thermodynamic parameters ΔH°, ΔS°, and ΔG°. The design expert program suggested 17 numerical possibilities for the maximum dye removal at the 99% desirability level using ANOVA within the experimental parameter range. The total cost of producing 1.0 g of AC(H) is estimated at 0.041 USD. These findings underscore the potential of AC(H) as a highly efficient adsorbent for MB removal, positioning it as a strong candidate for wastewater treatment applications.

**Supplementary Information:**

The online version contains supplementary material available at 10.1038/s41598-025-04240-9.

## Introduction

Environmental pollution poses a significant challenge across the globe because of social, technological, and industrial growth^1^. Water is the fundamental necessity for the survival of all living organisms^2^. It is estimated that 70–80% of illnesses in developing countries are linked to water contamination, with women and children being the most affected. As water pollution continues to cause widespread health issues, it has become a critical concern that requires urgent action to address and mitigate. The industrial wastes, including both toxic inorganic and organic pollutants, extremely contribute to water pollution when released into aquatic systems. Among these pollutants, the synthetic dyes, in accordance with their widespread application in industrial manufacturing facilities, become one of the most toxic water pollutants^1,3^. Methylene blue dye (MB) is a synthetic, heterocyclic aromatic compound, C16H18N3SCl 319.85 g/mol (3,7-bis(dimethylamino) phenothiazine chloride tetramethylthionine chloride), and cationic chemical compound^4^, widely employed in various industries as a cosmetic, in paper production^1^, rubber, plastic, leather processing, food, and pharmaceutical manufacturing^2^. The contaminated MB effluent from these industries has emerged as a significant environmental issue^5,6^. If MB is swallowed, it may irritate the digestive tract and produce nausea and vomiting, convulsions, shortness of breath, tachyarrhythmia, and blue disease. MB was reported as resistant to biodegradation due to its stable complex aromatic molecular structure containing a chromophore and polar groups^7,8^. So, to remove MB from effluent before releasing it into the aquatic environment, practical and innovative technologies are needed^9,10^. As a result, a wide range of biological, chemical, and physical methods should be utilized, such as chemical precipitation, reverse osmosis, nanofiltration, electrocoagulation, electrodialysis, ion exchange, and photocatalysis^11^.

But each of these strategies has its own limitations due to economic and technical reasons^12^. These reasons may include high cost, chemical consumption, heavy equipment, tricky handling, design, and operation^13^. Alternatively, adsorption, which is a well-known separation method, revealed excellent application in MB elimination with great competence^14,15^ and low costs^16,17^. Adsorption has several advantages over previous methods of electrochemical cell^1,18^, including the following: (a) efficiency in removing both carbon-based and inorganic pollutants, (b) accessibility, (c) manageability and simplicity, and (d) adsorbents can be easily restored and recycled.

Several forms of adsorbents, including carbon-based and natural polymeric materials, are considered the most efficient adsorbents. Activated carbon (AC) is the main significantly effective carbonaceous adsorbent^19^. Agricultural residue-based ACs provide eco-friendly sustainable materials with extraordinary removal proficiency for low production costs^20^. Such a policy can be counted as an invasive tool to minimize pollution of solid waste produced from agricultural crop harvesting and processing^20,21^. Agricultural wastes that are widely applied in AC production include fruit stones, bagasse, rice hulls, kenaf core fiber, nutshells, grape seeds, corncobs, sugar beet pulp, and leaves^22^.

For AC production, two protocols for activation are widely adopted: Physical and chemical procedures^23^. In the physical protocol, the starting precursor material is carbonized at elevated temperatures ranging from 500 to 900 °C in inert conditions. Then a successive cycle of activation is conducted for the obtained activated carbon at temperatures ranging from 800 to 1000 °C in CO_2_ or H_2_O vapor conditions as oxidizing gases or a mixture of them^24^. Conversely, the activation via chemical protocol encompasses the impregnation of the precursor materials in a suitable agent using inert conditions. The widely applicable activating agents include H_3_PO_4_ and ZnCl_2_, which act as dehydrating/pyrolytic chemicals, enhancing AC yield by^25^. For environmental and economic considerations, H_3_PO_4_ became the most acquainted agent in AC production in the last decade^26^.

It has been extensively researched how AC can be utilized to eliminate MB from textile wastewater^27^. For instance, MB was removed from wastewater using activated carbon, with a maximum removal effectiveness of 93.8%. Also, AC was developed from organics to remove MB from wastewater with a maximum removal efficiency of 99.9%^28^. Although CAC, or commercial-grade AC, has been applied for dye removal, its use is limited by its high cost and lack of regeneration properties^29^.

Egypt is one of the leading countries in the production of sugarcane crops, with approximately 325,000 hectares of cultivated land annually in Upper Egypt, where the warm climate supports high yields. Egypt’s annual production of sugarcane is about 16 million tons/year, with the primary use being sugar extraction^30^. A significant by-product of sugarcane processing is the fibrous residue, referred to as sugarcane bagasse (SCB), which consists of hull and core. This residue is abundant, comprising about 30% of the processed sugarcane by weight^30^. The hull and core are often burned as fuel for energy production or left as waste to be dumped^30^. However, these wastes hold great potential as raw materials for other applications, such as the production of bio-based adsorbents, paper, and biochar, due to their high lignocellulosic content. Thus, the efficient utilization of SCB waste in Egypt presents both a challenge and an opportunity for enhancing sustainability and reducing environmental impacts in the agricultural sector.

So, the current study aims to (1) recycle SCB wastes (hull and core) in ACs production via thermo-chemical protocol using H_3_PO_4_ as an activating agent; (2) Comparing the morphological, structural, and geometrical characteristics, as well as the removal efficiency of hull-derived (AC(H)) and core-derived ACs; (3) Evaluating the impact of some physicochemical experimental parameters upon the proficiency of MB remediation by AC(H) from synthetic solutions; (4) Elaborating the behavior and mechanism of MB sorption by setting the equilibrium data to familiar kinetic and isotherm models in both linear and non-linear forms; (5) Highlighting the thermodynamics’ characteristics of MB removal by AC(H); (6) Finally, evaluating the competence of AC(H) regeneration using a suitable reagent.

## Materials and methods

### Materials and chemicals

Sugarcane bagasse (SCB) waste (hull and core), the solid residue that remains after juice extraction, was employed in the current study as the basic raw material for active carbon (AC) preparation. This waste contains almost 20–24% lignin and 30–50% cellulose^31^. Such waste was collected from a small drinking stall in Beni-Suef Governorate, Egypt. Whereas phosphoric acid (H_3_PO_4_) (85%), which was employed as the chemical activating agent for both types of SCB waste (hull and core) to prepare AC via thermochemical activation protocol, was delivered from E. Merck, Germany. Methylene Blue, MB, dye with a molar mass of 319.85 g/mol and a C_16_H_18_N_3_SCl formula that was employed as a cationic contaminant in the current study, was secured by Fluka, Switzerland, and employed without further purification. To prepare a 1000 mg/L stock solution of MB, 1 g of MB was dissolved in 1000 mL of distilled water (DW). From this stock, the desired diluted concentrations for the subsequent experimental work were prepared using DW. The pH of these MB solutions was adjusted to the chosen values by adding diluted droplets of HCl (0.01 M) or NaOH (0.01 M).

### Preparation and chemical activation of sugarcane bagasse (SCB)

The SCB was washed with tap water to make sure that the SCB was free of any fine dust particles. Then the soft and smooth, pale fibers (hull, HSCB) were ripped off from the inner side of the SCB (core, CSCB) by hand. Those two samples were sanitized using distilled water (DW) and then placed separately in aluminum plates to be dried up at 70 °C/2 h till complete moisture removal. Each of the dried samples was shaped into small pieces using scissors (< 2 mm). Then a fixed weight of the bagasse fractions (core and hull), 10 g, was added separately to an equivalent weight of H_3_PO_4_, achieving a 1:1 w/w ratio to prepare two mixtures (hull/H_3_PO_4_ and core/H_3_PO_4_). Each mixture was liquefied with 100 mL DW and kept overnight with no interruption at ambient temperature. After excess acid decantation, the separated, soaked solid waste was washed by DW several times till pH neutralization. The separated solid fractions were then dried for 24 h at 100 °C, then weighed and calcined in open air at 700 °C (5 °C/min) for 2 h, using a programmable muffle furnace. Each prepared AC sample was gently ground using an agate mortar to get black powder (< 100 µm). The powder of each sample was stored in an airtight glass bottle as AC(H) and AC(C) to be characterized and further applied.

### ACs characterization

The XRD patterns (50°–80°) of the prepared ACs (AC(H) and AC(C)) were identified using a Shimadzu XRD-7000 diffractometer with a scanning speed of 2°/min and Cu K radiation at 40 kV/20 mA. Also, the structural analysis of these ACs was assessed using FT-IR in the range of 400–4000 cm^−1^ (Shimadzu IR-Tracer 100 with mode of reflection at a 4 cm^−1^ resolution). Whereas the morphological topography of these ACs was evaluated using SEM (JEOL JSM-6610 LV, Japan). On the other hand, the Vt (pore volume), Dp (average pore diameter), and SBET (BET surface area) of the addressed ACs were estimated via Surface Area Analyzer (Nova 2000)” after degassing at 373 K/180 min. The BJH equation (Barrett–Joyner–Halenda) was employed to determine Vt and Dp of these ACs^32^. Oppositely, the BET equation “Brunauer–Emmett and Teller” was employed to estimate the SBET of the discussed materials^33^.

### Batch studies

To elaborate on the ideal conditions for MB ion sorption by the prepared ACs in the batch system, the impact of several experimental factors was gauged: initial solution pH, AC mass, initial MB concentration, employed temperature, agitation time, and rate, as was illustrated in Table 1. For each experimental factor, the initial and residual MB ion concentrations in the supernatant at equilibrium were estimated using a UV–visible spectrophotometer (DR 6000) at λ “max” = 665 nm^20,21,34,35^. For accuracy, the experiments of each factor were conducted in triplicate, and the average result was recorded; at equilibrium, the removed amount (q_e_ or q_t_, mg/g) of MB ions by the applied ACs was estimated using Eqs. (1) and (2) orderly (Table 2). Whereas the uptake capacity (R%) of MB ions by the addressed ACs was defined via Eq. (3) (Table 2)^11,36^.

**Table 1:** The applied experimental parameters and the prevailing conditions during the conduction of MB sorption experiments by the prepared ACs.

Investigated parameter	Conditions								The other investigated parameters
pH	2	3	4	5	6	7	8	11	50 mg/L initial MB conc. in 25 mL solution, 4 mg dose of AC_(C)_ and AC_(H)_, 200 rpm/2 h (agitation time/speed) and temp. 298 K
Agitation time (min)	5	10	15	30	45	60	90	120	pH 9.0, 50 mg/L of MB initial conc.in 25 mL solution, 4 mg dose of AC_(H)_, 200 rpm/2 h (agitation time/speed) and temp. 298 K
AC_(H)_ dose (mg)	2	4	6	8	10				pH 9.0, 50 mg/L of MB initial conc. in 25 mL solution, 200 rpm/h (agitation time/speed) and temp. 298 K
MB initial conc. (mg/L)	10	20	30	40	50	60	70		pH 9.0, 4 mg dose of AC_(H)_, 25 mL solution, 200 rpm/h (agitation time/speed) and temp. 298 K
Temperature (K)	291	298	303	308	313	318	323	328	pH 9.0, 50 mg/L of MB initial conc. in 25 mL solution, 4 mg dose of AC_(H)_, 60 rpm/h (agitation time/speed)
Agitation rate (rpm)	50	100	150	200	250				pH 9.0, 50 mg/L of MB initial conc. in 25 mL solution, 1 h agitation time, 4 mg dose of AC_(H)_ and temp. 298 K

**Table 2:** Equilibrium equations that express the MB sorption by prepared ACs.

Equation no.	Linear form	Parameters
Equation 1	$q_{e} = \frac{{V\left( {C_{i} - C_{f} } \right)}}{m}$	*q*_*e*_ (mg/g): sorbed amount of MB at equilibrium *C*_*i*_: the initial MB concentration in solutions (mg/L) *C*_*f*_: the concentration of MB at equilibrium (mg/L) *V*: the volume of MB solutions (mL) *m*: the mass of adsorbent (mg)
Equation 2	$q_{t} = \frac{{V\left( {C_{i} - C_{t} } \right)}}{m}$	*q*_*t*_ (mg/g): sorbed amount of MB at time t *C*_*i*_: the initial MB concentration in solutions (mg/L) *C*_*t*_: the concentration of MB (mg/L) at time t *V*: the volume of MB solutions (mL) *m*: the mass of adsorbent (mg)
Equation 3	$R\% = \frac{{\left( {C_{i} - C_{t} } \right)}}{Ci} \times 100$	*R%*: removal efficiency of MB by addressed adsorbents *C*_*i*_: the initial MB concentration in solutions (mg/L) *C*_*t*_: the concentration of MB (mg/L) at time *t*

### Adsorption kinetics

The adsorption path and mechanism, as well as the adsorption rate, were analyzed and determined by applying the linear fitting of the pseudo-first-order, pseudo-second-order, and intraparticle diffusion and Boyd models for the experimental data evaluation, as shown in Table 3.

**Table 3:** Sorption kinetics models for MB uptake by AC_(H)_.

Kinetic model	Linear form	Nonlinear form	Parameters	Refs.
Pseudo-first order (PFO)	$\ln \left( {q_{e} - q_{t} } \right) = \ln q_{e} - k_{1} t$	$q_{t} = q_{e} \left( {1 - exp - {\text{K}}_{1} t} \right)$	*q*_*t*_ (mg/g): removed amount of MB at time *t* *q*_*e*_ (mg/g): equilibrium sorption uptake *k*_1_ (g/mg min): rate constant of the first-order adsorption	Lagergren and Svenska^37^
Pseudo-second order (PSO)	$\frac{t}{{q_{t} }}\, = \,\frac{1}{{k_{2} \,q_{e}^{2} }} + \,\frac{t}{{q_{e} }}$	$q_{t} = \frac{{k_{2} t\left( {q_{e} } \right)^{2} }}{{1 + k_{2} q_{e} t}}$	*q*_*t*_ (mg/g): removed amount of MB at time t *q*_*e*_ (mg/g): equilibrium sorption uptake *k*_2_ (g/mg min): rate constant of the second-order adsorption	Ho and McKay^38^
Intra-particle diffusion (IPD)	$q_{t} \, = \,k_{p} \,t^{1/2} \, + \,C$	–	q_t_ (mg/g): removed amount of MB at time t K_p_ (mg/g min^0.5^): intra-particle diffusion rate constant *C* (mg/g): intercept of the line which reflects the thickness of the boundary layer *k*_*p*_ = *slope* *C* = *intercept*	Weber and Morris^80^

### Adsorption isotherm

The equilibrium investigation was applied to indicate the interaction between the adsorbed metal ions and the used adsorbent by fitting the experimental equilibrium data to the linearized and non-linearized forms of Langmuir, Freundlich, Temkin, and Dubinin–Radushkevich (D–R) isotherm models, as shown in Table 4.

**Table 4:** Sorption isotherm models for MB uptake by AC_(H)_.

Isotherm model	Linear form	Non-linear form	Parameters	Refs.
Langmuir	$\frac{{C_{e} }}{{q_{e} }} = \frac{1}{{q_{\max } b}} + \frac{{C_{e} }}{{q_{\max } }}$ $R_{L} = 1/\left( {1 + bC_{0} } \right)$ $R_{L}$ > 1 (unfavorable adsorption) $R_{L}$ = 1 (linear adsorption) $0 < R_{L}$ < 1 (favorable adsorption) $R_{L}$ = 0 (irreversible adsorption)	${\text{q}}_{{\text{e}}} = \frac{{{\text{q}}_{\max } {\text{b C}}_{{\text{e}}} }}{{1 + {\text{b C}}_{{\text{e}}} }}$	C_e_ (mg/L): equilibrium concentration of the residual MB in the solution q_e_ (mg/g): removed amount of MB at equilibrium q_max_ (mg/g): maximum adsorption capacity b (L/mg): Langmuir constant C_0_: Initial MB concentration R_L_: Equilibrium parameter of Langmuir equation	Langmuir^107^
				Weber and Chakravorti^39^
Freundlich	$\log q_{e\,} = \,\,\log k_{F} + \,\frac{1}{n}\,\log \,C_{e}$	${\text{q}}_{{\text{e}}} { } = {\text{KF}}\left( {{\text{C}}_{{\text{e}}} 1/{\text{n}}} \right)$	C_e_ (mg/L): equilibrium concentration of the residual MB in the solution q_e_ (mg/g): removed amount of MB at equilibrium k_F_ (mg/g): MB adsorption capacity n: heterogeneity factor	Freundlich^40^
Temkin	${\text{q}}_{{\text{e}}} = {\text{B}}\ln {\text{A}} + {\text{B}}\ln {\text{C}}_{{\text{e}}}$ $B = \,\,RT/b$	${\text{q}}_{{\text{e}}} { } = {\text{B ln}}\left( {{\text{AC}}_{{\text{e}}} } \right)$ $B = \,\,RT/b$	A (L/g): Temkin isotherm constant (the equilibrium binding constant corresponding to the maximum binding energy) B (J/mol): Temkin constant related to heat of sorption b: Temkin isotherm constant (slope) R: The gas constant (8.314 J/mol K) T : the absolute temperature at 298 K	Tempkin and Pyzhev^41^
Dubinin–Radushkevich (D–R)	Ln ${\text{q}}_{{\text{e}}}$ = Ln q_max_ − Kε^2^ ε = RTLn(1 + 1/C_e_)	${\text{q}}_{{\text{e}}} = {\text{q}}_{{{\text{max}}}} {\text{exp}}\left( { - {\text{K}}\upvarepsilon ^{2} } \right)$ $\upvarepsilon = {\text{RTln}}\left( {1 + \frac{1}{{{\text{C}}_{{\text{e}}} }}} \right)$ ${\text{E}}_{{{\text{DR}}}} = \sqrt {\frac{1}{{2{\text{K}}_{{{\text{DR}}}} }}}$		Dubinin and Radushkevich^106^

### Adsorption thermodynamics

The nature, spontaneity, and interior energy change of the adsorption of MB onto the AC_(H)_ surface was determined by the evaluation of the thermodynamic parameters, including the Gibbs free energy (ΔG, kJ/mol), enthalpy (ΔH, J/mol), entropy (ΔS, J/K mol), and the activation energy (Ea) by the Van’t Hoff expression, as shown in the following equations^19^.

4 $$\therefore \Delta {\text{G}}^{\circ } = \Delta {\text{H}}^{\circ } - {\text{T}}\Delta {\text{S}}^{\circ }$$

5 $$\Delta {\text{G}}^{\circ } = - {\text{RT}}\ln {\text{K}}_{{\text{d}}}$$

6 $${\text{K}}_{{\text{d}}} = {{{\text{q}}_{{\text{e}}} } \mathord{\left/ {\vphantom {{{\text{q}}_{{\text{e}}} } {{\text{c}}_{{\text{e}}} }}} \right. \kern-0pt} {{\text{c}}_{{\text{e}}} }}$$

7 $$\ln {\text{K}}_{{\text{d}}} = \frac{{\Delta {\text{S}}^{\circ } }}{{\text{R}}} - \frac{{\Delta {\text{H}}^{\circ } }}{{{\text{RT}}}}$$

where R is the universal gas constant (8.314 J/mol K), T is the absolute Kelvin temperature, and K_d_ is the thermodynamic equilibrium constant (distribution coefficient).

### Experimental design and data analysis

In this work, response surface methodology (RSM) employing the CCD technique was used to analyze data and create experiments using Create-Expert software (version 8.0.0). A statistical technique called RSM makes use of quantitative data from studies to investigate how several parameters impact the process and how different components respond when they are varied simultaneously. Contact time (A), pH (B), adsorbent dosage (C), and starting concentration (C) are the four primary independent variables that were optimized, and their effects on MB removal utilizing the AC(h) design (3-level-4-factor) were investigated in this work. Table S1 lists the levels and range of variables that influence the removal efficiency of MB. These variables include low level (− 1), central (0), and high level (+ 1). Through statistical evaluation of the *P* value and F-value of regression coefficients (*P* < 0.05), analysis of variance (ANOVA) was used to examine the impact of input factors on the response.

Furthermore, adequate accuracy (AP), adjusted coefficient of determination (R^2^ adj.), and coefficient of determination (R^2^) were used to report the model’s validity. Lastly, to illustrate the relationship between independent elements and their associated impacts on the response, three-dimensional response level diagrams have been created. As recommended by DOE (version 8.0.0) software, 29 runs (three repeats) of the tests were conducted. Table S2 displays the average of every run, except for the 6 central runs. The expected response was computed using the quadratic equation model (Eq. 8), which incorporates all interaction factors^42^.

8 $${\text{Y}} =\upbeta 0 + \sum\upbeta {\text{iXi + }}\sum {\text{i}}\sum {\text{j}}\upbeta {\text{ijXiXj}} +\upvarepsilon$$

The coefficients of the statistical model were evaluated using the quadratic model based on Eq. (3) whenever Y is the response, β0 is the constant coefficient (the intercept), βi is the linear coefficient, βij is the interaction coefficient, βii is the quadratic coefficient, xi and xj are the coded values of the variables under investigation, and e is the statistical error term.

## Results and discussion

### Characterization of the prepared active carbons (ACs)

**Fig. 1:**
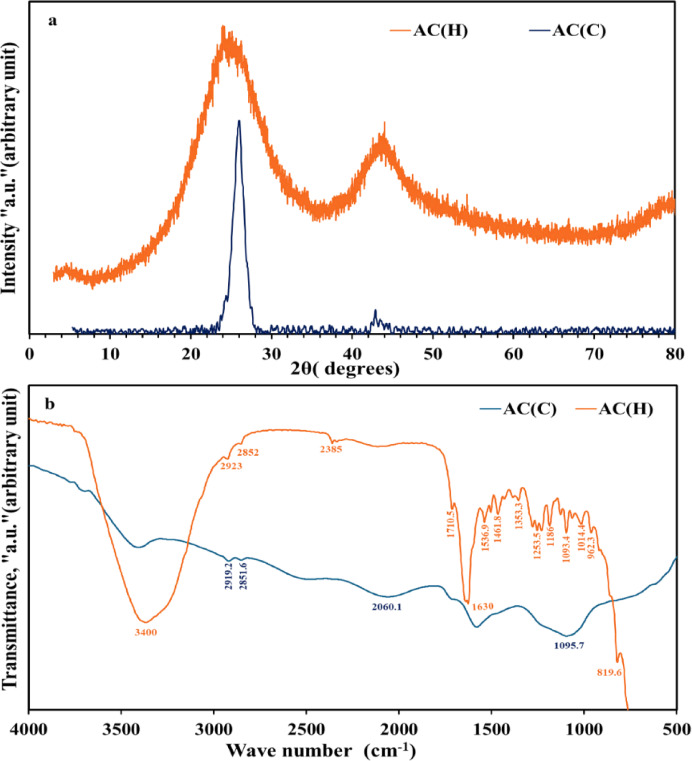
XRD patterns (**a**) and FT-IR spectra (**b**) of AC_(C)_ and AC_(H)_.

The XRD patterns of the produced ACs from both the hull and core of the SCB wastes via chemo-thermal activation protocol were compiled in Fig. 1a. The broad diffraction hump that was observed in AC(H)’s pattern at 2θ ≈ 20°–30° could be attributed to γ-phase carbon of AC, suggesting a more disordered carbon structure with a higher degree of porosity^43,44^. Such an amorphous hump was probably inherited from the amorphous parental material, SCB, hull^20,21,45–47^. The broader peak in AC(H) implies a more developed microporous structure compared to AC(C). However, in the AC(C) pattern, such a hump was overprinted with a very sharp diffraction peak at 2θ ≈ 25.98° with d spacing 3.43 Å, such peaks due to amorphous SiO_2_^46–49^. This could indeed be associated with the biogenic silica that can be encountered in a higher ratio in the core than in the hull sample of SCB^50,51^. Despite the higher ashing degree in AC(C), some activated carbon with developed porosity could still be formed due to phosphoric acid chemical activation. This employed acid not only facilitated the creation of pores but also the formation of some activated carbon before extensive combustion occurs, leaving behind inorganic materials (biogenic silica) as ash.

On the other hand, broad diffraction peaks at 2θ ≈ 42°–46° in both AC patterns in favor of AC(H), could potentially be correlated to the structural features such as turbostratic ordering or stacking of carbon layers within the activated carbon structure^20,44^. Furthermore, the noticeable variation in ash content in both ACs in favor of AC(C), could be attributed to variations in the composition and structure of the initial raw materials (hull and core) used for each AC production. The core of SCB might contain a higher concentration of inorganic metals, such as silica, compared to the hull. These metals contribute to the ash content after the thermal treatment. Also, the variations in the proportions of lignin, cellulose, and hemicellulose between the core and hull can affect the yield of activated carbon and ash; the core might have higher lignin content, leading to more residue and ash upon decomposition. In accordance with variation in composition and structure between hull and core, their reactivity toward phosphoric acid might differ, affecting the extent of mineral removal during activation. The less reactivity of the core retains more inorganic material and hence higher ash content after thermal treatment. Thus, it can be concluded that the played role of phosphoric acid and the employed thermal protocol upon the activation process of SCB samples into ACs is critical^20^. Whereas the acid not only functioned as a dehydrating factor that influenced the pyrolytic decomposition of the hull and core of the SCB samples but also hampered ash formation and increased obtained carbon in AC(H)^52^.

The FTIR spectra of ACs prepared from both the hull and core of SCB are depicted in Fig. 1b. For AC(H), derived from sugarcane hull, the broad band at 3400 cm^−1^ corresponding to O–H stretching vibrations indicates the presence of hydroxyl and/or N–H stretching vibration groups, possibly due to residual cellulose or hemicellulose^53–55^. Additionally, bands around 2923 cm^−1^ and 2852 cm^−1^ are indicative of C–H stretching in aliphatic hydrocarbons^56^. Whereas the prominent band at 1710 cm^−1^ can be ascribed to C=O stretching in carbonyl groups, likely resulting from oxidation during the activation process. Moreover, the strong band at 1630 cm^−1^ is most likely due to C=C stretching in aromatic rings^20^. On the other hand, the bands between 1536.9 and 1253.5 cm^−1^ were associated with aromatic C=C and C–O stretching, orderly, suggesting a more complex and developed carbon structure^57^. Furthermore, the band at 819.6 cm^−1^, indicating aromatic C–H out-of-plane bending, points to well-developed aromatic rings in AC(H). The region between 1186 and 962 cm^−1^ includes bands corresponding to C–O stretching, indicating the presence of oxygenated functional groups that contribute to the adsorption properties of AC(H) by providing sites for chemical interactions, such as hydrogen bonding or dipole interactions, with sorbates. These functional groups are likely remnants of the original biomass material that have undergone transformation during the activation process^58^. In contrast, the FT-IR spectrum of AC(C), derived from the SCB core, shows a reduced intensity of the O–H and/or N–H groups around 3400 cm^−1^, reflecting their lower presence, likely due to increased ashing^59^. Similarly, the bands at 2919.2 cm^−1^ and 2851.6 cm^−1^ correspond to C–H stretching but are less intense than in AC(H), suggesting a reduction in aliphatic hydrocarbon content. The band around 1575 cm^−1^ in AC(C) also indicates C=C stretching in aromatic structures, highlighting some retention of aromatic character, although less pronounced than in AC(H)^20^. The approximate absence of the carbonyl band at 1710 cm^−1^ and the reduced intensity of aromatic C=C and C–O bands imply a less developed carbon structure in AC(C), aligning with the higher ash content observed in this sample. This is supported by the prominent band at 1095 cm^−1^, characteristic of Si–O–Si stretching^60,61^, which indicates the presence of silica, likely biogenic in origin. The increased ash content in AC(C) corresponds to a higher concentration of inorganic components, such as silica, which is consistent with the XRD findings and reflects the differences in the initial composition of the sugarcane core compared to the hull.

**Fig. 2:**
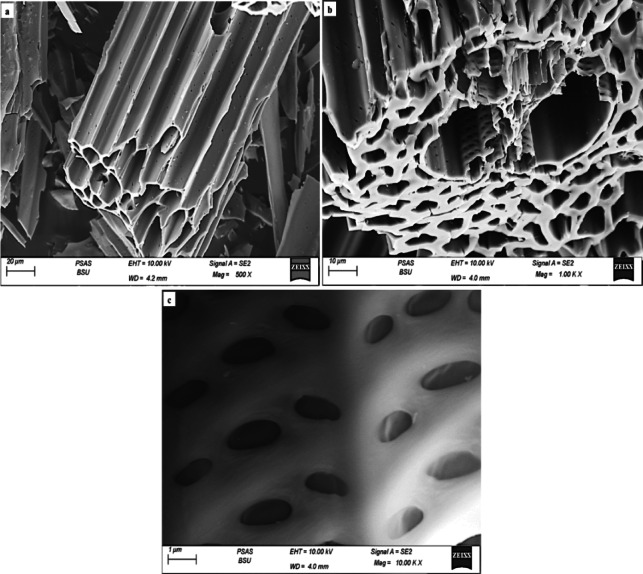
SEM images showing porous structure of AC_(H)_ at different magnifications (**a**–**c**).

The SEM images of AC derived from the hull of sugarcane bagasse (AC(H)) revealed several key characteristics. The morphology is notably heterogeneous, reflecting the mixed and fibrous nature of the hull material (Fig. 2a–c). The pore structure is varied, featuring a mix of micro- and mesopores with variable sizes and shapes, indicative of the less uniform pore distribution typical of hull-derived activated carbon^50,55^. The surface texture appears rough and inconsistent, likely due to the diverse components of the hull. Similarly, the displayed layering and wrinkles due to the arrangement of carbon atoms in sheets or layers (Fig. 2a,b) suggest the presence of semi-graphitic structures formed during the activation process, but with insufficient order and crystallinity to be traced using XRD patterns.

**Fig. 3:**
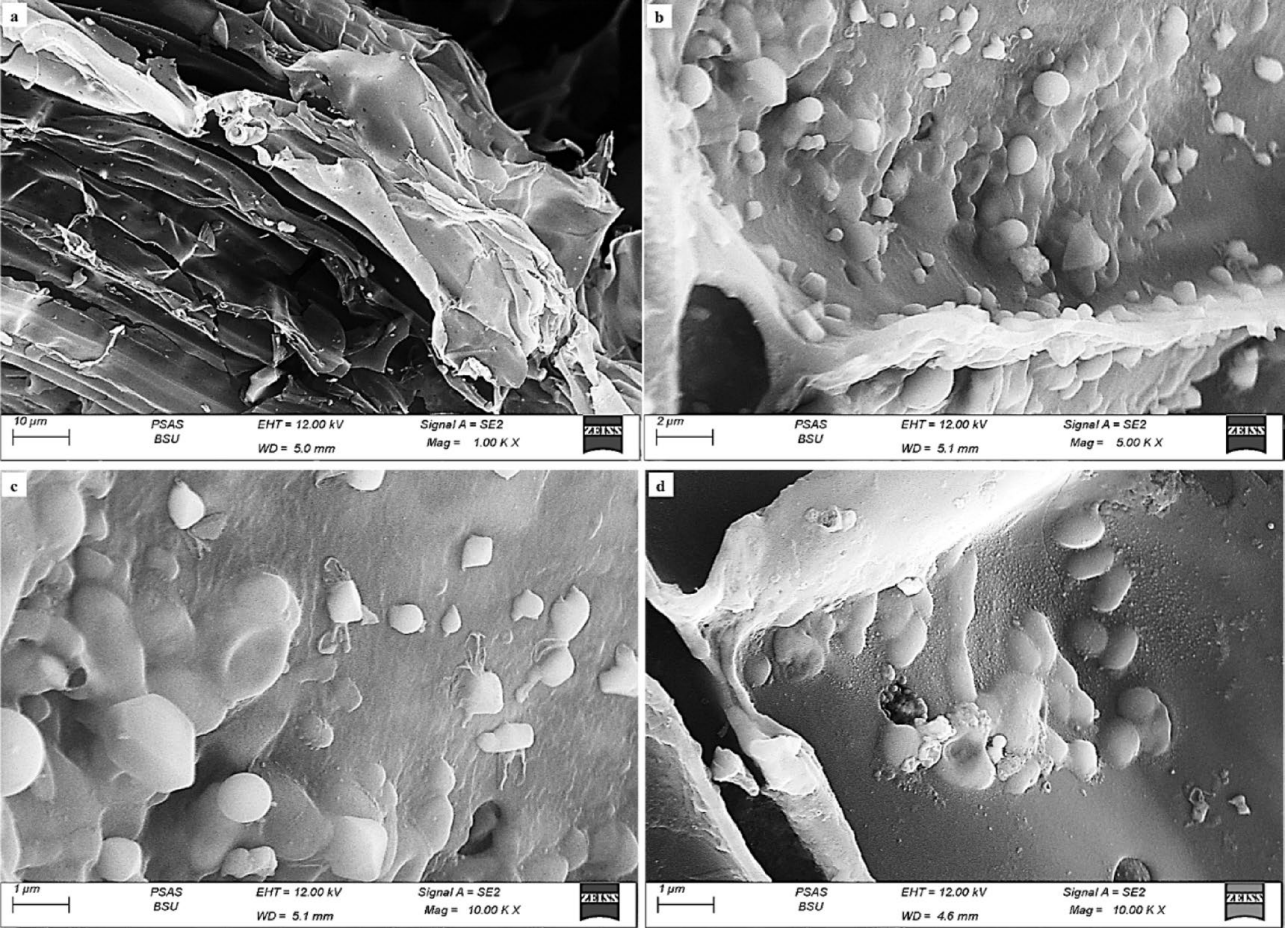
SEM images showing smooth morphological characteristic of AC_(C)_ (**a**) with prominent presence of biogenic silica in form of droplets and spheroidal particles at different magnifications (**b**–**d**).

The layering structure in the form of folded sheets which relate to the inner layer of hull bagasse provides more surface area for interactions, while the wrinkles create additional pores and voids of variable diameters within AC_(H)_, enhancing its porosity. Conversely, AC derived from the core of SCB (AC_(C)_) is notably less heterogeneous in morphology with an almost smooth surface texture (Fig. 3a) compared to AC_(H)_. This could be ascribed to the approximately homogenous nature of the precursor core material.

However, the pores’ structure is a less varied mix of micro and mesopores, but smaller in pore diameter compared to those of AC_(H)_. Additionally, the displayed layering and wrinkles are less developed as a sign of the very poor arrangement of carbon atoms in sheets or layers and hence the poor development of semi-graphitic structures during the applied activation processes (Fig. 3a). However, a prominent presence of biogenic silica in the form of droplets and spheroidal particles of various degrees of maturity and sizes was observed lining the layering structure of AC_(C)_ (Fig. 3b–d), aligning with XRD data.

**Fig. 4:**
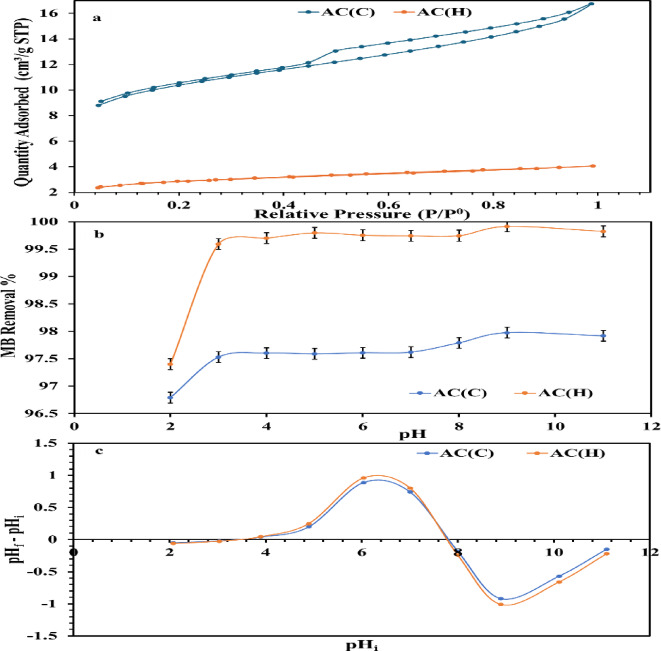
Nitrogen adsorption–desorption isotherms of AC_(C)_ and AC_(H)_ (**a**); Effect of pH on MB ions sorption by AC_(C)_ and AC_(H)_ (**b**) and point of zero charge of AC_(C)_ and AC_(H)_ (**c**).

The N_2_ adsorption–desorption isotherms for both activated carbons, AC_(C)_ and AC_(H)_ reveal distinct porosity characteristics as depicted in Fig. 4a. The isotherm of AC_(C)_ exhibits a type IV profile with a broad the hysteresis loop, indicative of mesoporous materials^62^. The broadening of the hysteresis loop and the overlapping of the adsorption and desorption branches suggest a complex pore structure with slit-like pores, which contributes to AC_(C)_’s greater total pore volume of 0.318 cm^3^/g and an average pore diameter of 1.50 nm (Table 3), making it suitable for adsorbing larger molecules. This wide range of pore diameters was supported by the incapability to reach N_2_ equilibrium status^20^. Conversely, the AC_(H)_ isotherm displays characteristics of a type II isotherm with hysteresis loop of H_3_ (Fig. 4a), which is indicative of the presence of both microporous and mesoporous structures^62,63^. This suggests the existence of thin and broader slit-like mesopores, which enhance the material’s adsorption capacity^64^. The initial section of the isotherm at low P/P^0^, where adsorption and desorption branches are overlapping, suggests the presence of micropores, where adsorption occurs primarily due to monolayer coverage^20^. As the relative pressure increases, the isotherm transitions into a linear region with slightly broadening hysteresis loop, indicative of multilayer adsorption on the exterior surface of the pores^20,21^. This behavior aligns with the BET surface area of 424.332 m^2^/g, the total pore volume of 0.288 cm^3^/g, and the average pore diameter of 1.36 nm (Table 5). High BET and pore size distribution make AC_(H)_ effective in adsorbing various molecules, particularly those that fit within the range of the presented pore sizes^43,65^. Despite the similar surface areas of both ACs, the differences in pore volume and average pore diameter underscore the impact of the raw material’s structural composition, core versus hull, on the final pore structure post-activation. Overall, the unique pore and surface area characteristics of both AC_(C)_ and AC_(H)_, suggest their potential versatility in various adsorption applications.

**Table 5:** Textural parameters of the prepared ACs obtained from the nitrogen adsorption/desorption isotherms.

Sample	SBET (m^2^/g)	Total pore volume (V_t_) (cc/g)	Average pore diameter (nm)
AC_(C)_	423.792	0.318	1.50
AC_(H)_	424.332	0.288	1.36

### Batch studies

#### Effect of pH

Applied initial pH played an essential role in MB uptake by the prepared ACs (AC(C) and AC_(H)_) from synthetic solutions (Fig. 4b). It was revealed that both ACs have remarkable removal efficiency for MB ions, with some privilege of AC(H) over AC(C) (R% = 96.79–97.98% and 97.4–99.14%, orderly) all over the examined pH range, 2.0–11.0 (Table 1). However, the maximum R% was accomplished at pH 9.0 for both adsorbents (R% = 97.98 and 99.14%, respectively). Such a finding aligns with the pHpzc (point of zero charge) of both ACs (pHpzc ≈ 7.8) estimated following the proposed protocol by^66^ (Fig. 4c). Conversely, the least removal % for MB ions by the addressed materials was achieved at an extremely acidic medium, pH 2.0 (R% = 96.79 and 97.4%, orderly), matching with pervious investigations^20,35^. Beyond pH 9.0, a slight decline in MB ion removal % was noticed by both adsorbents (97.92 and 99.83% for AC(C) and AC(H), consecutively), implying that equilibrium was attained^34^. At pH < pHPZC, the surfaces of ACs become steadily protonated with medium acidity rising. This resulted in an invasive repulsive reaction among the + ve MB ions and oxygen/nitrogen groups of the employed ACs. Conversely, at pH > pHPZC, the domination of − ve charges upon the surfaces of ACs due to deprotonation processes triggers the hydrolyzed species of MB+ ions to be attracted by these sites through electrostatic mechanism^67,68^. This highlights the effective role of electrostatic forces in remediating MB ions out of solutions via employed ACs especially at pH > pHPZC^69^.

The remarkable removal percentage of MB ions at the prevailing acidic conditions (acidic medium, pH 2.0–6.0), in spite of the violent H+ ion competition by both ACs, signifies that non-hydrophobic forces (i.e., electrostatic attraction) couldn’t be considered as the only driving mechanism for MB ions; other forces can be involved^70^. These hydrophobic forces (H-bonding) probably performed an essential part in the MB sorption by the studied materials^35,71^. The inherited hydrophobicity of these ACs from their organic contents is the probable source of such forces^71,72^. These hydrogen bonding connections can be defined as: (1) Yoshida and (2) dipole–dipole^35,73^. The interconnection among nitrogen/oxygen of MB ions (H-acceptor) with the available H (H-donor) of the OH groups upon the surfaces of the addressed ACs expresses the 1st H-bonding sort^35,73^. Conversely, the bonding between MB aromatic rings with OH groups of the addressed ACs signifies the 2nd H-bonding sort^35,70^. In light of the pH experimental work and the obtained geometrical data, XRD, and FT-IR results of the investigated ACs, pH 9.0 and AC(H) were selected for removal of MB ions from synthetic solutions in the upcoming experimental work (Table 1).

#### Effect of contact time

**Fig. 5:**
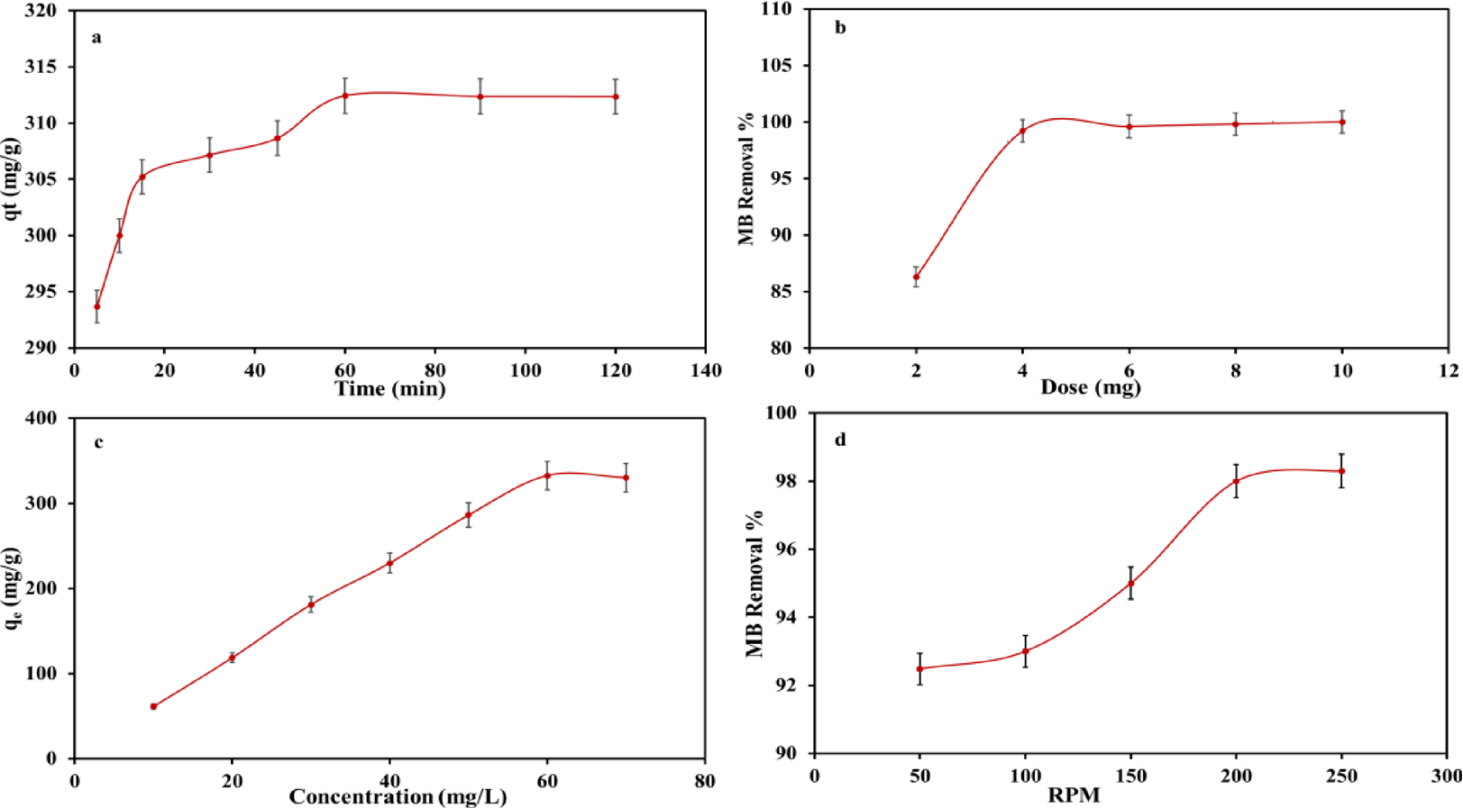
Effect of retention time, dose, concentration and rpm on MB ions sorption by AC_(H)_ (**a**–**d** respectively).

The remediation of MB ions from synthetic solution at variable retention time (5–120 min) by AC_(H)_, keeping the other experimental parameters constant, was found to be a time-dependent procedure (Fig. 5a, Table 1). This was accentuated through the very rapid remediation of MB ions at times ranging from 5 to 15 min (293.7–305.2 mg/g, orderly)^74^, aligning with previous investigations^35^. Such sorption behavior was ascribed to the approachability of vacant deprotonated groups on the AC_(H)_ surface with high affinity for MB ions^75^. Conversely, at equilibrium time 15 > t ≤ 60 min, the remediation rate of MB from standard solutions was decelerated (q_t_ ≈ 305.2–312.4 mg/g). Such an attitude could be linked to the drop in the accessible unoccupied sites on the AC(H) surface with time progress (Fig. 5a). Further than the equilibrium time of 60 min, a trivial change in the remediated MB ions by the addressed AC (q_t_ ≈ 312.3 mg/g) was observed. Accordingly, such adequate equilibrium presents AC_(H)_ as a favorable material for MB removal via the sorption process of a chemical nature. Hence, 60 min was assigned as MB sorption equilibrium time by AC_(H)_ in the succeeding experimental work (Table 1).

#### Effect AC_(H)_ mass

The remediation of MB ions using variable masses of the prepared AC_(H)_ (2–10 mg) was thoroughly investigated, maintaining the other employed parameters fixed (Fig. 5b, Table 1). It was revealed that the increase in the employed mass of AC_(H)_ from 2 to 4 mg was coupled with a noticeable development in MB removal % from 86.3 to 99.2%, orderly. However, a further rise in the employed dose from 6 to 8 mg was supplemented by a marginal increase in R% from 99.6 to 99.8%, implying the attainment of equilibrium status^20^. But the utilization of 10 mg of AC_(H)_ was enough to achieve a complete MB removal with R% 100%. This was ascribed to the huge availability of free deprotonated sites (N- and O-holding groups) on AC_(H)_ with high affinity toward MB ions^76,77^.

#### Effect of initial concentration

The applied initial concentration of sorbate has a critical impact upon the sorption process and consequently the R% of the investigated material^34^. To configure this impact, numerous initial concentrations of MB sorbate (10–70 mg/L) were utilized, preserving the constancy of the other involved experimental parameters (Fig. 5c, Table 1). With raising MB concentration from 10 to 60 mg/L, the sorbed amount (q_e_) of MB ions was steadily improved from 61.3 to 332.6 mg/g, successively, per employed dose of AC_(H)_. Such remarkable development in the absorbed amount of MB ions could be tied with the ascending rate of MB ions collision with the available free sites on the AC surface^20,35^. The acceleration in the rate of collision among the MB ions at higher initial concentration enforces these ions toward the active sites upon adsorbent’s surface, facilitating their attraction and remediation by AC_(H)_^78^. However, further progress in the employed initial concentration beyond 60 mg/L (i.e. 70 mg/L) was accompanied by marginal change in the removed amount of MB ions (q_e_ ≈ 330.1), signifying equilibrium status. This can be justified by the unavailability of free active sites on the AC_(H)_ surface (i.e. sites becoming saturated) to be involved in the sorption process of MB ions, aligning with several reported data^20,35^.

#### Effect of agitation rate

To illustrate the critical role of the applied agitation speed upon the sorption process of MB ions^43,79^ by the investigated AC_(H)_, various agitation rates (50–250 rpm) were cautiously examined, maintaining the other experimental factors constant (Fig. 5d, Table 1). The progressive increase in the employed agitation speed from 50 to 200 rpm was coupled with an appreciable improvement in the MB R% from 92.48 to 98% (Fig. 5d). However, further progress in the employed agitation speed to 250 rpm was accompanied by marginal improvement in the achieved MB R% (98.4%). This signifies that 200 rpm was enough rate to achieve equilibrium status for MB remediation by the investigated AC_(H)_. So, this agitation rate was utilized in conducting the other experimental investigations.

#### Kinetics investigations

**Fig. 6:**
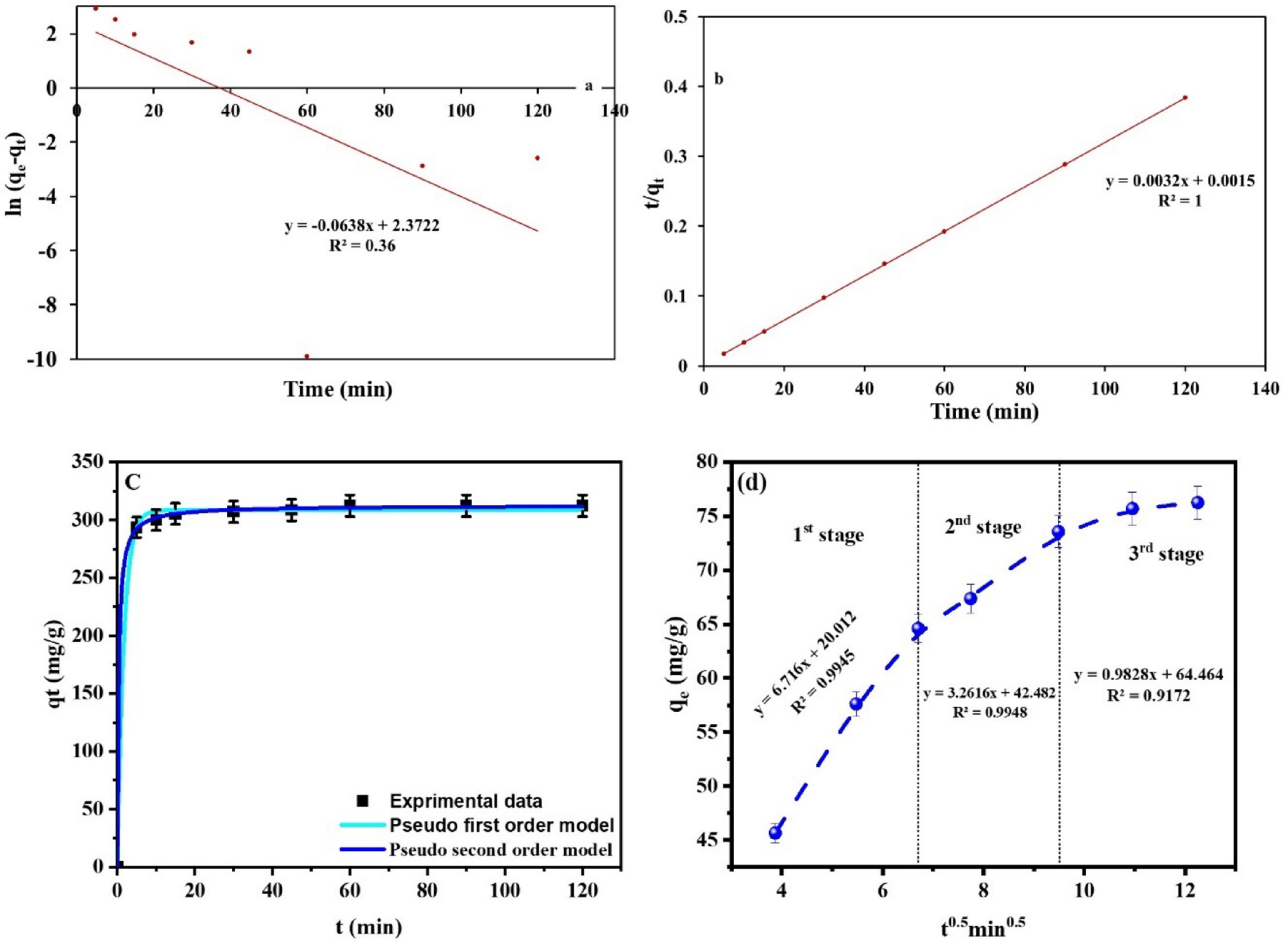
Linear and non-linear plots of PFO and PSO (**a**–**c**), orderly, as well as IPD (**d**) kinetic models for MB sorption by AC_(H)_.

To spell out the mechanism of MB ion sorption by the investigated AC_(H)_, three of the most widely applicable equations in this field were employed: pseudo-1st order (PFO), pseudo-2nd order (PSO), and intra-particle diffusion. The linear and non-linear mathematical forms of both PFO^37^ and PSO^38^ models were employed (Table 4). Whereas the linear expression of the IPD^80^ was applied in the current study, using a t^0.5^ vs. q_t_ plot (Fig. 6d, Table 6). To determine PFO and PSO parameters for the linear equations, t vs. ln(q_e_ − q_t_) and t vs. t/q_t_ plots were employed, orderly (Fig. 6a,b). While the fitting to the non-linear curve t vs. q_t_ (Fig. 6c) was utilized to define the parameters of the non-linear expressions of these equations by the means of Microsoft Excel Solver Tool^35^.

**Table 6:** Kinetic parameters of the MB sorption by AC(H).

Model	Variable	Unit	Linear	Non-linear				
PFO	q_e_^(exp.)^	(mg/g)	312.4	312.4				
	q_e_	(mg/g)	10.72	308.55				
	K_1_	(min^−1^)	0.064	0.596				
	R^2^		0.36	0.998				
PSO	q_e_	(mg/g)	312.5	312.36				
	K_2_	(g/mg min)	0.0068	0.009				
	R^2^		1	0.9998				
IPD	K_P_	(mg/g min^0.5^)	3.515	3.515				
	C	(mg/g)	37.46	37.46				
	R^2^		0.898	0.898				

Step 1			Step2			Step3		
k_P_1	C_1_	R^2^	k_P_2	C_2_	R^2^	k_P_3	C_3_	R^2^
Linearized Interparticle diffusion model								
6.716	20.012	0.9945	3.2616	42.482	0.9948	0.982	64.464	0.9172

In light of R^2^ (regression determination coefficient) outcomes, it was revealed that the sorption process of MB by AC_(H)_ was justified by PSO, in both linear and non-linear forms, in a more fluent way than PFO (Fig. 6a,c). This implies that during the MB sorption process by AC_(H)_, chemisorption may be the rate-driving force^43,81^. Such a conclusion was based on the astonishing R^2^ for both linear and non-linear regression fitting of PSO (R^2^ = 1 and 0.9998, orderly) that exceeded those of the PFO equation (R^2^ = 0.36 and 0.998, orderly), especially that of the linear expression^34^. This was also enforced by the identical matching among the experimental (q_eexp_ = 312.4 mg/g) and calculated q_e_ values (q_ecal_ = 312.5 and 312.36 mg/g) that were derived from linear and non-linear regressions, successively (Table 5)^20^. Such matching was not achieved by the PFO, especially the linear regression fitting (q_ecal_ = 10.72 and 308.55 mg/g for linear and non-linear mathematical expressions, respectively) (Table 5).

Furthermore, to elaborate on the nature of MB ion diffusion from the synthetic solution toward the AC_(H)_ surface, the IPD model was employed. The obtained graphical presentation of the IPD model was constructed of 3 different steps, indicating its multilinearity and deviation from the point of origin (Fig. 6d). Thus, intra-particle diffusion was not the main diffusion type in MB sorption process^68^. However, several types of diffusions may be included^82^. The 1st step (steep section at time interval 0–15 min of contact time) of the multi-linear plot expresses that boundary layer diffusion or exterior mass transfer was the main diffusion process that caused a very fast MB sorption by the de-protonated sites on the AC_(H)_ surface^20,83^. As the sorbent material molecules diffuse from the solution, they move toward the material’s outside surface under investigation^84,85^. This very rapid sorption process was ascribed to the plentiful unoccupied groups on the AC_(H)_ surface and the higher concentration of MB ion in the solution at such contact time^86^. Oppositely, the 2nd gently inclined step at time interval ≥ 15 t ≤ 60 min, whereby the molecules penetrate the materials’ pores^84,85^, expresses the domination of intra-particle diffusion during the gradual sorption process of MB ions by the active functional groups of the investigated AC_(H)_^75^. Meanwhile, the boundary diffusion layer thickness reflected by the high C value (297.91 mg/g) demonstrates the efficient participation of the AC(H) surface in MB removal. At such time intervals, the deceleration in the MB sorption process can be linked to the decline in the number of vacant binding groups on the AC_(H)_ surface and the reduction in the MB ion concentration in the solution with time, as well as the longer diffusion pathway of MB ions into the inner surface and pores of^40,41,87^ AC_(H)_^20,86^. This was followed by the plateau stage (3rd step beyond 60 min of contact time), expressing nearly a fixed rate of MB sorption by AC_(H)_ due to equilibrium attainment^88^. As the boundary layer value rises across the stages, indicating an increase in the molecules adsorbed on the material’s surface, the kid values decrease throughout the stages, corresponding to the adsorption process gradually slowing down^84,85^.

Therefore, the 2nd step of this multi-linear plot was selected to determine IPD parameters, KP (297.91 mg/g min^0.5^) and C and R^2^ (0.918) (Table 6).

#### Isotherm investigations

**Fig. 7:**
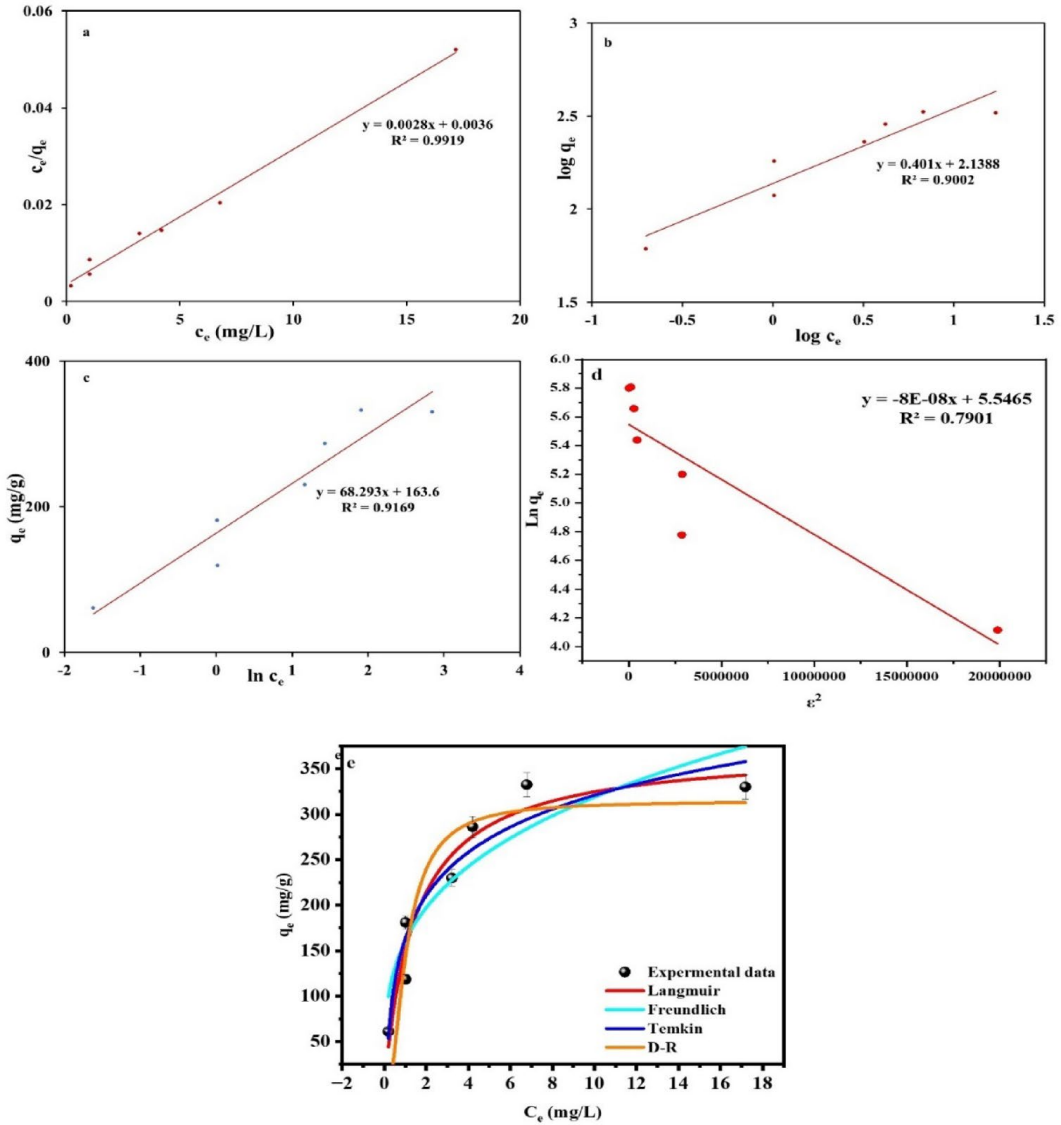
Linear (**a**–**d**) and non-linear (**e**) plots of Langmuir, Freundlich, Temkin and DR.

To elaborate the interaction nature of the investigated MB sorbate and the binding sites of the AC_(H)_, the linear and non-linear mathematical expressions for three of the widely applicable isotherm models, Langmuir, Freundlich, and Temkin, were employed (Table 7). From the linear expressions of these models, these models’ parameters were estimated using c_e_ vs. c_e_/q_e_, log c_e_ vs. log q_e_, ln c_e_ vs. q_e and ε_ plots, orderly (Fig. 7a–d, Table 7). Furthermore, to estimate the parameters of the nonlinear expressions of these equations, c_e_ vs. q_e_ nonlinear plot was employed, using the Solver tool of Microsoft Excel (Fig. 7e, Table 7). Langmuir equation presumes a monolayer sorption process of the sorbate ions form the solution via distinct active groups on the adsorbent surface with iso-energetic power^35,89,90^. Conversely, Freundlich pretends to be the heterogeneous multilayer sorption theory of sorbate ions upon adsorbent surface by aniso-energetic functional groups^91^.

**Table 7:** The isotherm parameters of the MB adsorption by AC_(H)_.

Model	Parameter	Linear form	Non-linear form
		Value	Value
Langmuir	q_max_ (mg/g)	357.14	389.4
	b (L/mg)	0.778	0.549
	R^2^	0.992	0.973
	R_L_	0.018–0.114	–
Freundlich	1/n	0.401	0.278
	K_F_ (mg/g)	137.66	170.29
	R^2^	0.90	0.929
Temkin	B (J/mol)	36.28	65.93
	A (L/g)	10.97	13.85
	R^2^	0.917	0.961
D–R	K_D_	8E-08	1.65
	q_max_ (mg/g)	256.34	314.51
	E (kJ/mol)	2500	550.8
	R^2^	0.79	0.863

Unlike the Langmuir and Freundlich equations, Temkin equation accounts specifically for the indirect sorbate-sorbate interactions effects. It also points toward the fact that the sorption heat declines linearly instead of logarithmically with coverage, implying that as more sorbate ions are absorbed, the energy required for further sorption declines^86^. The DR isotherm is an empirical isotherm model that describes the degree of surface covering features and the adsorption mechanism with Gaussian energy distribution. Experimental data was further confirmed by distinguishing between chemical and physical adsorption of adsorbents using the DR model. Chemisorption was suggested by mean free energy (E) more than 8.00 kJ/mol. The preponderance of chemisorption is further supported by the activation energy (EDR) of 550.8 and 2500 J/mol in non-linear and linear, respectively, which suggests that the MB and the functional groups in the sorbent material have formed chemical bonds.

Considering the determination coefficient (R^2^) results of the three employed equations in both modes, linear and non-linear forms, Langmuir (R^2^, 0.992 and 0.973, orderly) justified MB sorption data by the investigated AC_(H)_ in sufficient way than Temkin (R^2^, 0.917 and 0.961, orderly) and Freundlich (R^2^, 0.900 and 0.929, orderly) models. This implies that the sorption process of MB ions had a homogeneous nature. This nature was expressed by the formulation of singular-layer of MB ions through the attraction forces of iso-energetic binding groups on AC_(H)_ surface^20,69^. Similarly, estimated R_L_ (0.018–0.114) using the equation compiled in Table 6^39^, that lies within the limit 0 < RL < 1, indicates favorable sorption process and confirms the Langmuir pertinency to elucidate clearly the sorption process of MB ions by AC_(H)_^35^.

Furthermore, the expectational q_max_ (357.41 and 389.4 mg/g from linear and non-linear equations, respectively) for the MB sorption process at equilibrium aligns with the high S_BET_ and unique porous structure of the investigated AC_(H)_ (Table 3). It also designates the extreme affinity of the deprotonated − ve functional groups on the AC_(H)_ surface toward the MB ions^20,92^.

For comparison, the displayed q_max_ of the investigated AC_(H)_, was compiled with those of some other adsorbents of previous studies that were used also for MB remediation (Table 8). In the light of such comparison, AC_(H)_ may be designated as a sustainable, cost-wise, and competent adsorbent for MB remediation on an industrial scale.

**Table 8:** Adsorption capacity of MB by some natural, modified and synthetic materials in comparison with the AC_(H)_ of the present study: q_max_ obtained from the Langmuir constant.

Adsorbent	Adsorption capacity q_max_ (mg/g)	References
An/CNT composite	416.7	^43^
Zeolite/chitosan composite	199	^93^
Sericin/β-cyclodextrin/PVA composite	261	^94^
Halloysite-cyclodextrin nanosponges	226	^95^
Cotton fiber	113	^96^
Modified pine sawdust	84	^97^
Fe-MIL-88NH_2_	15.99	^98^
Poly(*N*-isopropyl acrylamide-coitaconic acid)/crosslinked with OVPOSS	130	^99^
Carboxylmethyl cellulose coated Fe_3_O_4_@SiO_2_ magnetic nanoparticles	17.5	^100^
Ephedra strobilacea char (ESC)	31.055	^75^
Phosphoric acid modified Ephedra strobilacea char (ESP)	21.88	^75^
Zinc chloride modified Ephedra strobilacea char (ESZ)	37.174	^75^
Talc-graphite schist	9.41	^35^
C-RWB	43.7	^64^
AC-alg membrane	666	^1^
AC_(H)_	357.14 (linear) 389.4 (non-linear)	Current study

#### Thermodynamic investigations

Temperature performances a vital role in the sorption processes of certain sorbate by specific adsorbent as it controls both the capacity and the rate of sorption. The temperature manipulation in the kinetic energy of pollutant molecules, their movement in solution and hence solid–liquid interface, justifies such critical impact of temperature on sorption capacity and rate of sorption^86^.

**Fig. 8:**
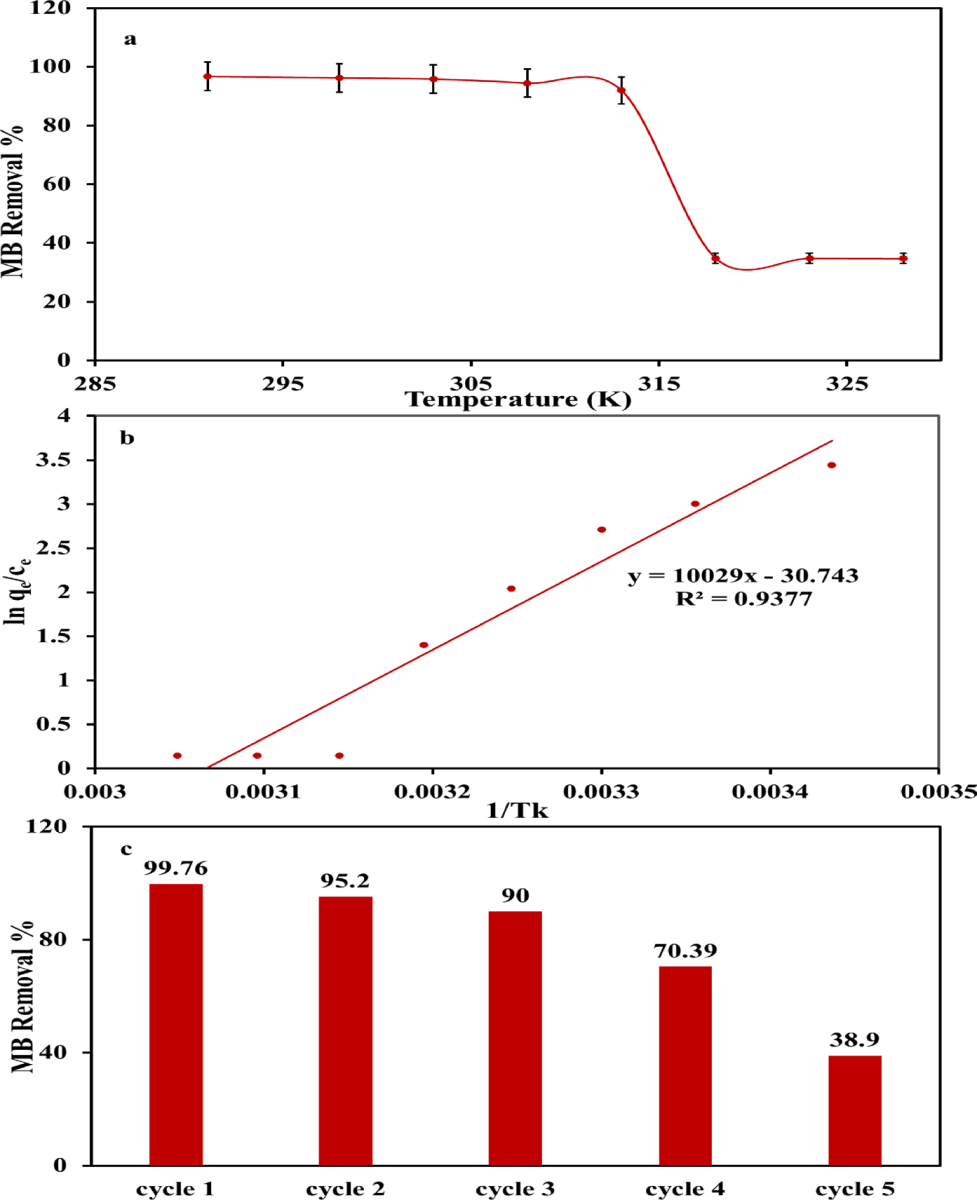
Effect of temperature on MB removal by AC_(H)_ (**a**); Van’t Hoff plot (**b**); and Reusability test of MB sorption by AC_(H)_ (**c**).

Also, temperature can modify the surface characteristics of the employed adsorbent, such as the availability and activity of functional groups, which in turn affects the sorption efficiency. For instance, the dissociation or association of surface groups can be temperature dependent. Therefore, configuring the vital role of temperature not only allows for better optimization of sorption systems, but also can improve sorption efficiency, selectivity, and the operational life of the adsorbent^35^. In this context, the removal% profile of MB ions from synthetic solutions at different temperatures (291–328 K) was cautiously studied (Fig. 8a, Table 1). The MB removal% by AC_(H)_ was relatively declined at temperature interval 291–313 K, with values ranging from 96.7 to 91.92%. This indicates that the MB sorption process is highly effective within this temperature range, likely due to optimal interactions between the MB ions and the binding groups on the AC_(H)_ surface. However, the slight reduction in removal% suggests that the MB sorption process is not significantly affected by temperature variations within this range, possibly indicating a low activation energy for the sorption process. Conversely, a sharp decline in MB removal% was observed as the temperature increased > 313 K, dropping to around 35% (Fig. 8a). This drastic decline implies that the MB sorption by AC_(H)_ is an exothermic process^86^ unlike several reported data^34,35^. As the temperature rises, the shifting in equilibrium may lead to this decline in sorption capacity as the system favors desorption over adsorption. This sensitivity to temperature variation consists of the exothermic nature of many sorption processes where higher temperatures reduce the interaction between the sorbate and the adsorbent, possibly due to increased kinetic energy leading to desorption of previously adsorbed MB molecules^86^. According to Le Chatelier’s assumption, temperature increase shifts the equilibrium to favor desorption. The stabilization of removal% around 35% beyond 320 K indicates that the sorption sites of AC_(H)_ are no longer as effective at higher temperatures. This could also imply that some structural changes of AC_(H)_ and/or MB might occur at these elevated temperatures, further reducing the R%^86,101^. For better conceptualization of the close relation between the prevailing solution temperature and MB sorption process by AC_(H)_. The plot of ln K_d_ vs. 1/T (Fig. 8b) is a fundamental tool in understanding the thermodynamics of the MB sorption process by AC_(H)_. In this context, q_e_ expresses the amount MB ions absorbed at equilibrium, whereas c_e_ is the concentration of dye at equilibrium, and T is the prevailing temperature in Kelvin. The linearity displayed by such plot confirms that the MB sorption process by the addressed AC_(H)_ obeys Van’t Hoff equation that links the constants of sorption equilibrium to temperature. Intercept and slope of Van’t Hoff plot (Fig. 8b), were employed to determine the values of ΔS° and ΔH°, orderly (Table 9). The negative ΔH° value (− 83.38 kJ/mol) suggests that the MB sorption process is exothermic^86^. Similarly, the − ve ΔS° value (− 0.256 kJ/mol K), suggests that MB sorption process results in a decline in the randomness/disorder at the liquid–solid interface (i.e., the system becomes more ordered)^34^. This improvement in system order was probably correlated with the alignment of MB ions upon AC_(H)_ surface, matching with the above-mentioned homogeneous monolayer sorption of Langmuir via isoenergetic sites. Moreover, the negativity of ΔS° value also implies that MB sorption process may involve a more structured interaction with AC_(H)_, indicating a drop in the overall entropy of the system.

**Table 9:** Thermodynamic parameters of MB sorption process by AC_(H)_.

∆S° (kJ/mol K)	∆H° (kJ/mol)	∆G° (kJ/mol)							
		291 K	298 K	303 K	308 K	313 K	318 K	323 K	328 K
− 0.256	− 83.38	− 8.884	− 7.092	− 5.812	− 4.532	− 3.252	− 01.972	− 0.692	0.588

Regarding ΔG° outcomes for all the investigated temperatures (Table 9), the − ve values at lower temperatures imply that the MB sorption process by AC_(H)_ is spontaneous and thermodynamically favorable^34,86,91^. However, as the temperature increases, ΔG° approaches zero and eventually becomes positive (0.588 kJ/mol at 328 K), demonstrating that the process becomes less spontaneous at higher temperatures; positive ΔG° value, indicates non-spontaneous sorption process. This shift reflects the exothermic manners of the sorption process, where the driving forces decrease with increasing temperature, ultimately leading to a less favorable sorption at higher temperatures. The ΔG° range of values that lies approximately between 0 and − 20 kJ/mol, except for 328 K, implies that the mechanism of MB sorption process was physisorption, primarily due to weak Van Der Waals forces or electrostatic interactions^86^. As well, this ΔG° range indeed supports the idea that the sorption of MB on AC_(H)_ is primarily driven by π–π interactions and hydrogen bonds^86^. These interactions are consistent with the observed thermodynamic properties, indicating a strong but reversible process that aligns with physical sorption enhanced by specific, non-covalent interactions.

The possible number of adsorption–desorption cycles of the regenerated AC_(H)_ was recorded (Fig. 8c) as reusability test. It was revealed that the investigated AC_(H)_ retains a high uptake competence, where the MB R% still surpassed 70% after four consecutive cycles before the noticeable decline to 38.9% at the 5th cycle. This exhibits that the AC_(H)_ can be counted as cost-effective and recyclable adsorbent for the MB remediation^102^.

#### Process analysis and modeling of the MB removal

Multiple regression analysis was utilized to examine the association between the response value and four parameters: the response time, adsorbent dosage, initial pH, and initial MB concentration. Table 4 provides an overview of the analysis of variance (ANOVA) results. pH, contact time, beginning dye concentration, and adsorbent dosage all showed a favorable impact on the removal effectiveness of MB, according to the analysis of the F and *P* values of the variables examined in this study. Furthermore, AC, BD, AD, and CD all had *P* values below 0.05, indicating a significant impact on MB sorption. The first dye concentration and adsorbent dosage have the biggest impact on dye removal.

A strong correlation between the predicted and experiment results is confirmed by the adjusted R_2_ and correlation coefficient (R_2_) for the MB removal, which are 0.978 and 0.9663, respectively.

However, the model was significant for MB removal, as indicated by the Model F-value (180.84) whereas it is recorded 58.5^103,104^. Prob > F values below 0.05 are interpreted as indicating that the model terms were significant, whilst values over 0.05 suggest that the model terms are not. The model’s sufficiency is confirmed by the non-significant absence of ft value 6.54. Signal to noise ratio value of 4 or higher is typically considered needed for adequate precision^103^ whereas it is recorded 3.53^103^. The model developed in this work may be used to explore the design space since the achieved appropriate precision for the deterioration of MB was 44.539, whereas it recorded 29.69653^103^, which verified an adequate signal (Eq. 8). Y = 92.14 + 4.32 A + 3.98 B + 6.87 C + 2.62 AB + 2.93 AC − 0.1245 BC − 2.14 A2 − 2.22 B2 − 4.73 C2.

**Fig. 9:**
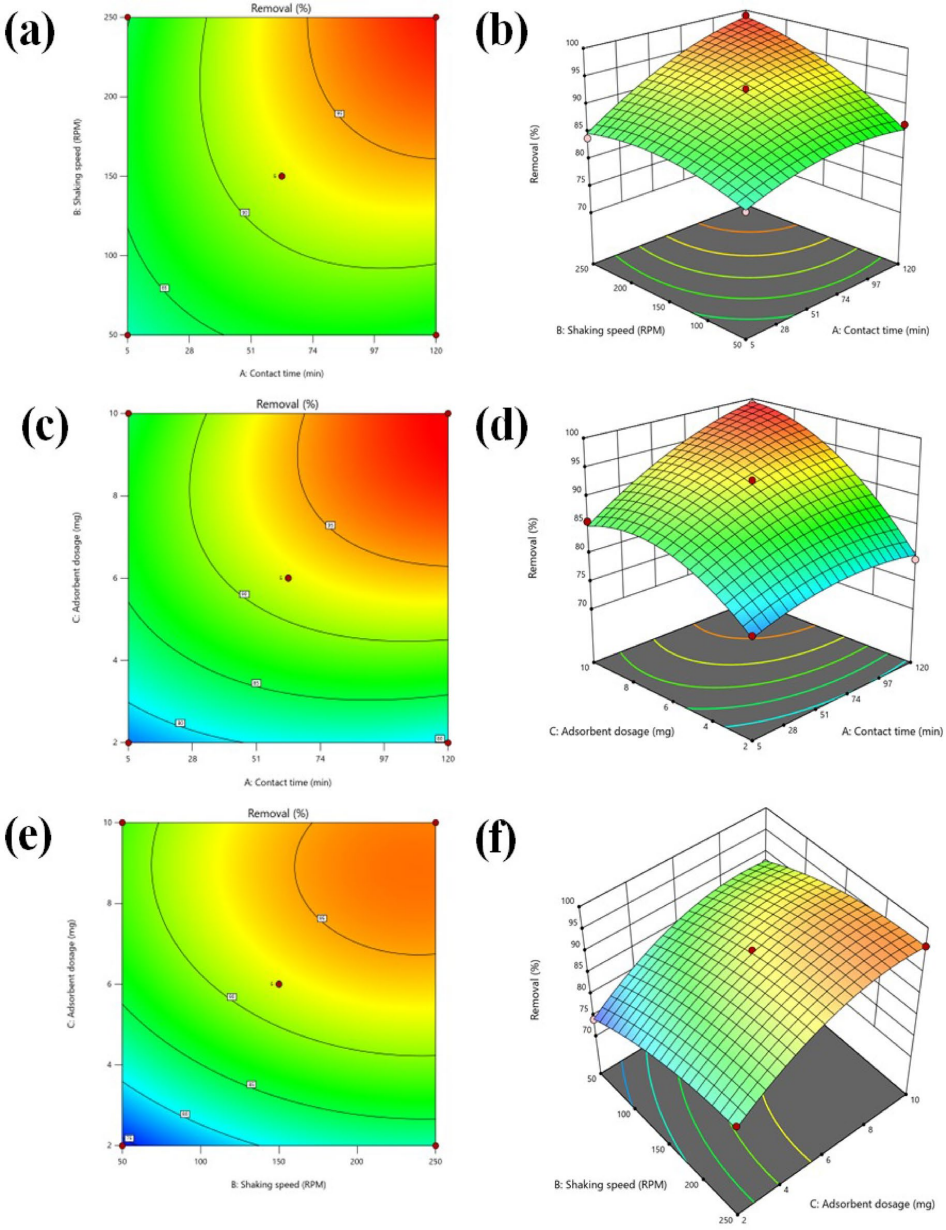
(**a**) 2D response surface and (**b**) 3D contour plots show the effect of AC_(H)_ dose on shaking speed; (**c**) 2D response surface and (**d**) 3D contour plots AC_(H)_ dose and contact time 2D response surface and (**d**) 3D contour plots AC_(H)_ dose and shaking speed on the removal of MB (%).

Figure 9 displays the AC_(H)_ elliptical response surface plot. Dosage and duration of contact for the elimination of MB sorption by AC_(H)_. The effectiveness of dye removal and contact time with RPM were shown to be significantly impacted by the interaction between dosage and RPM. The percentage of dye elimination rose when the contact duration was extended from 5 to 120 min. Near the response surface’s center points, maximum removal was achieved. At a dose of 10 mg and a duration of 120 min, respectively, a maximum MB sorption of 99% was anticipated; contact lasted 90 min.

Within the experimental parameter range, Design Expert software recommended 17 numerical options for the highest dye removal at the 99% desirability level. Under the following conditions, the proposed model’s numerical solution projected the maximum dye removal (99%): 200 RPM, 90 min of contact time, and a dose of 10 mg of adsorbent.

#### Regeneration investigations

The competence of sorbent materials to be regenerated and recycled several times is an important aspect as it affects production expenses^34^. The regeneration of the spent AC_(H)_ was carried out using HCl (5%) solution, invasive distilled water washing and oven-drying at 70 °C.

#### Cost analysis

The economic viability of an adsorbent plays a crucial role in determining its practical applicability for wastewater treatment. The total preparation cost comprises expenditures on energy consumption and chemical inputs. As summarized in Table 10, the total cost of producing 1.0 g of AC_(H)_ is estimated at 0.02 USD.

**Table 10:** Cost analysis of the synthesized composite material.

Material	Purchased quantity (g/mL)	Total purchase cost (USD)	purchasing cost (USD/mL)	Used quantity (mL)	Cost of used quantity (USD)
Sugarcane bagasse (SCB) waste	500	–	–	200	–
Phosphoric acid (H_3_PO4) (85%)	1 L	3.00	0.003	200	0.6

Equipment	Time (H)	Max. power (kW)	Unit cost of power	Cost	
Drying	26	1	0.24	6.24	
Calcination	2	1	0.36	0.72	
		Total yield cost = 7.56 USD For 182.38 g	Total yield cost 0.041 USD/g		

#### Future study

Future research should concentrate on increasing the preparation of AC(H) for continuous-flow treatment systems in order to more accurately replicate industrial settings, building on the encouraging findings of this study. Its field application will be further confirmed by examining its adsorption behavior in actual wastewater matrices that contain organics, heavy metals, or mixed colors. Sustainability can also be improved by investigating green regeneration techniques (such as solar-assisted desorption or microwave). To increase selectivity for particular contaminants, AC_(H)_ surface modification or functionalization may also be investigated. Finally, employing this biosorbent in hybrid treatment systems (such as AOPs + adsorption) could pave the way for extremely effective and reasonably priced wastewater cleanup.

## Conclusions

On the light of the present work outputs, the following deductions can be represented; The malignant environmental impact of SCB wastes can be eliminated through their conversion into sustainable and cost-wise ACs via thermo-chemical protocol (H_3_PO_4_/SCB, 1:1 w/w ratio at 700 °C/2 h). The produced ACs exhibited distinct structural and textural characteristics, with the hull-derived AC (AC_(H)_) showing a more developed micro/meso-porous structure and higher S_BET_ (424.332 m^2^/g) compared to the core-derived AC (AC_(C)_, S_BET_ = 423.792 m^2^/g). The appreciable presence of biogenic silica in the form of droplets and spheroidal particles of various degrees of maturity and sizes in AC_(C)_, indicates a higher ash content in this product that can be correlated to the noticeable inorganic content in the precursor core. This was supported by the prominent existence of Si–O–Si group at 1095 cm^−1^ absorption band in AC_(C)_ spectra. The MB sorption process by AC_(H)_ was a pH/time liable practise; the maximum R% was accomplished at pH 9.0 and 60 min as equilibrium time. The kinetics of MB sorption onto AC_(H)_ were expressed well by PSO equation, indicating that chemisorption was the inspiring mechanism. This was enforced by the very close agreement between the linear and nonlinear regression analyses (R^2^ > 0.999) and the astonishing coincidence between the experimental (q_e exp._ = 312.4 mg/g) and theoretical q_e_ values (q_e calc._ = 312.5 and 312.36 mg/g for linear and non-linear regressions, orderly). Several intervening diffusion styles including IDP can be counted as the inspiring step in MB sorption process upon AC_(H)_ surface_._ Linear and nonlinear isotherms demonstrated that MB sorption data were elucidated in a fluent way using Langmuir equations, with very high R^2^ values > 0.97 in both regression modes than those of Freundlich and Temkin equations. This supports the homogenous accumulation assumption of MB ions as a monolayer via N-and O-bearing iso-energetic functional groups on the AC_(H)_ surface. Thermodynamic parameters, ΔH°, ΔS°, and ΔG°, verified the spontaneous and exothermic style of MB sorption process by AC_(H)_ at 291–323 K; the sorption process becomes less spontaneous at higher temperatures of this range. Beyond this temperature range (> 323 K), the positive ΔG° value (0.588 kJ/mol at 328 K), indicates a non-spontaneous MB sorption process; the driving forces decrease with increasing temperature, ultimately leading to a less favorable sorption at higher temperatures. A strong correlation between the predicted and experiment results is confirmed by the adjusted R_2_ and correlation coefficient (R^2^) for the MB removal, which are 0.978 and 0.9663, respectively.

Regeneration investigations disclosed that spent AC_(H)_ could be recycled till the 4th cycle (R% > 70%) before the appreciable decline in its removal efficiency. Finally, AC_(H)_ can be classified as a long-term sustainable solution for SCB wastes elimination and as an eco-friendly cost-wise alternative to commercially activated carbons that used in thiazine dyes remediation form wastewater.

With the first-ever utilization of sugarcane bagasse hull and core, this study presents a novel method for creating tailored activated carbon that outperforms several current biomass-based adsorbents in terms of methylene blue adsorption capacity, reaching up to 357.14 and 389.4 mg/g) in linear and non-linear modes, respectively. The study further distinguishes itself by utilizing Design Expert software to combine adsorption isotherms, kinetics, and thermodynamic studies with ANOVA-based optimization—a technique that is rarely used in similar work. An effective and sustainable adsorbent is created by the economically and environmentally sustainable activation process, which uses easily accessible agricultural waste^105^. Its practical potential is shown by the results, which demonstrate great adsorption performance with R^2^ values surpassing 0.97 under optimum conditions. However, the study is constrained by its single dye testing and its emphasis on batch processes rather than continuous flow systems.

## Electronic supplementary material

Below is the link to the electronic supplementary material.

Supplementary Material 1

## Data Availability

Data is provided within the manuscript.
