# Supplementary material for: Statistical modeling of mutagenic azo dye adsorption on bagasse activated carbon
Source: Sci Rep. 2025 Jun 20;15:20112. doi: 10.1038/s41598-025-04240-9 (PMC12181272; doi:10.1038/s41598-025-04240-9)
Supplement: Supplementary file 1 — Supplementary Material 1 [file 41598_2025_4240_MOESM1_ESM.docx]

**Statistical Modeling of Mutagenic Azo Dye Adsorption on Bagasse Activated Carbon**

**S_1_ :Table 1** Independent variables and their levels for designing the experiments used in the present study

| Parameters Name | Symbol | Unit | Low | Mid | High |
| --- | --- | --- | --- | --- | --- |
| Initial pH | A | - | 2 | 6.5 | 11 |
| Contact time | B | min | 5 | 62.5 | 120 |
| Initial concentration | C | mg/l | 10 | 40 | 70 |
| Adsorbent dosage | D | mg | 2 | 6 | 10 |

**S1:Table 2** Experimental conditions for adsorption process in the presence of prepared AC(H)

| Run No. | Parameters | | | | | | | | R% |
| --- | --- | --- | --- | --- | --- | --- | --- | --- | --- |
|  | Coded | Factor A | Coded | Factor B | Coded | Factor C | Coded | Factor A |  |
| 1 | 0 | 2 | 0 | 62.5 | -1 | 10 | 0 | 6 | 96.02 |
| 2 | 0 | 2 | 0 | 62.5 | 0 | 40 | -1 | 2 | 85.895 |
| 3 | 0 | 2 | +1 | 120 | 0 | 40 | 0 | 6 | 89.857 |
| 4 | +1 | 11 | 0 | 62.5 | +1 | 70 | 0 | 6 | 77.45714 |
| 5 | 0 | 6.5 | 0 | 62.5 | +1 | 70 | -1 | 2 | 70.4571 |
| 6 | 0 | 6.5 | 0 | 62.5 | 0 | 40 | 0 | 6 | 91.96 |
| 7 | 0 | 6.5 | 0 | 120 | 0 | 40 | +1 | 10 | 92.3 |
| 8 | 0 | 6.5 | -1 | 62.5 | +1 | 70 | +1 | 10 | 75.951 |
| 9 | 0 | 6.5 | -1 | 62.5 | 0 | 40 | 0 | 6 | 91.96 |
| 10 | +1 | 11 | +1 | 120 | 0 | 40 | 0 | 6 | 93.14 |
| 11 | -1 | 2 | 0 | 62.5 | 0 | 40 | +1 | 10 | 87.678 |
| 12 | +1 | 11 | 0 | 62.5 | 0 | 40 | -1 | 2 | 90.89 |
| 13 | 0 | 6.5 | +1 | 120 | +1 | 70 | 0 | 6 | 74.87 |
| 14 | 0 | 6.5 | 0 | 62.5 | -1 | 10 | -1 | 2 | 94.87 |
| 15 | 0 | 6.5 | 0 | 62.5 | 0 | 40 | 0 | 6 | 91.96 |
| 16 | -1 | 2 | -1 | 5 | 0 | 40 | 0 | 6 | 83.898 |
| 17 | 0 | 6.5 | 0 | 62.5 | 0 | 40 | 0 | 6 | 91.96 |
| 18 | 0 | 6.5 | +1 | 120 | -1 | 10 | 0 | 6 | 99.02 |
| 19 | 0 | 6.5 | -1 | 5 | +1 | 70 | 0 | 6 | 69.964 |
| 20 | 0 | 6.5 | 0 | 62.5 | 0 | 40 | 0 | 6 | 91.62 |
| 21 | -1 | 2 | 0 | 62.5 | +1 | 70 | 0 | 6 | 73.963 |
| 22 | 0 | 6.5 | -1 | 5 | 0 | 40 | +1 | 10 | 87.84 |
| 23 | 0 | 6.5 | -1 | 5 | 0 | 40 | -1 | 2 | 83.654 |
| 24 | +0 | 6.5 | +1 | 120 | 0 | 40 | -1 | 2 | 89.874 |
| 25 | +1 | 11 | -1 | 5 | 0 | 40 | 0 | 6 | 85.964 |
| 26 | 0 | 6.5 | 0 | 62.5 | -1 | 10 | +1 | 10 | 98.854 |
| 27 | +0 | 11 | 0 | 62.5 | -1 | 10 | 0 | 6 | 99.01 |
| 28 | 0 | 6.5 | -1 | 5 | -1 | 10 | 0 | 6 | 96.84 |
| 29 | +1 | 11 | 0 | 62.5 | 0 | 40 | +1 | 10 | 94.98 |

**S_3_:Table 3**: ANOVA results for the regression equation describing MB removal efficiency as a function of the variables studied

| source | Sum of squares | df | Mean square | *F* value | *p* value |  |
| --- | --- | --- | --- | --- | --- | --- |
| Model | 2013.5 | 14 | 143.82324 | 81.86 | < 0.0001 | Significant |
| A | 1679.192 | 1 | 1679.19185 | 955.70 | < 0.0001 | Significant |
| B | 79.573 | 1 | 79.57265 | 45.29 | < 0.0001 | Significant |
| C | 48.522 | 1 | 48.522 | 27.62 | 0.0001 | Significant |
| D | 40.197 | 1 | 40.197415 | 22.88 | 0.0003 | Significant |
| AB | 1.86 | 1 | 1.857769 | 1.06 | 0.3213 |  |
| AC | 0.064 | 1 | 0.06354 | 0.0362 | 0.8519 |  |
| AD | 0.57 | 1 | 0.56995 | 0.3244 | 0.5780 |  |
| BC | 0.37 | 1 | 0.3703 | 0.2107 | 0.6532 |  |
| BD | 0.774 | 1 | 0.7744 | 0.4407 | 0.5175 |  |
| CD | 1.331 | 1 | 1.3306 | 0.7573 | 0.3989 |  |
| A^2^ | 146.664 | 1 | 146.66353 | 83.47 | < 0.0001 | Significant |
| B^2^ | 33.19 | 1 | 33.19 | 18.89 | 0.0007 | Significant |
| C^2^ | 4.368 | 1 | 4.3676 | 2.49 | 0.1372 |  |
| D^2^ | 14.7743 | 1 | 14.7743 | 8.41 | 0.0116 | Significant |
| Resuidal | 24.598 | 14 | 1.75702 |  |  |  |
| Lack of fit | 24.506 | 10 | 2.4506 | 105.99403 | 0.0002 | Significant |
| Pure error | 0.0925 | 4 | 0.02312 |  |  |  |
| Cor total | 2038.124 | 28 |  |  |  |  |
